# Surgical mask covering of N95 filtering facepiece respirators: The risk of increased leakage

**DOI:** 10.1017/ice.2021.50

**Published:** 2021-02-09

**Authors:** Jeffrey T. Mueller, Soroor Karimi, Karl A. Poterack, Maria Teresa A. Seville, Steven M. Tipton

**Affiliations:** 1 Mayo Clinic College of Medicine and Science, Phoenix, Arizona; 2 University of Tulsa, Tulsa, Oklahoma

In this report, we demonstrate the potential risk of increased face-to-mask seal leakage when N95 filtering facepiece respirators (N95 FFRs) are covered by surgical, cloth, or medical masks, (collectively referred to as surgical masks), through analytical modeling of the associated fluid mechanics and seal pressures. Previously published experimental studies of respirator pressures and leakage are applicable to this problem. Properly utilized N95 FFRs will remain an essential component of healthcare worker safety for the foreseeable future, especially for those engaged in aerosol-generating procedures (AGPs) such as endotracheal intubation.^1–3^ When considering leakage risk, it is important to understand the general challenges to ensuring an adequate mask-to-face seal. The fit and seal degrade with repeated donning and doffing, and some N95 FFR reprocessing or recycling techniques have been reported to accelerate this degradation.^4^ In short, the N95 mask-to-face seal is fragile and can be compromised by a number of factors.

## Methods

The surgical mask creates additional resistance to airflow compared to only an N95 FFR. As a porous medium, the ease of movement of the fluid through the N95 can be modeled as permeability *“k”* in Darcy’s law.^5^ Darcy’s law states that the flow rate through the porous medium is proportional to the permeability and the pressure drop across this medium:(1)
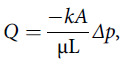



where *Q* is the volumetric flow rate (analogous to minute ventilation), *A* is the cross-sectional area, *µ* is fluid viscosity, *L* is the length of the porous medium, and Δ*p* is the pressure drop.^5,6^ This law states that for the same amount of pressure drop, the flow rate permeating the porous medium decreases as the length of the medium increases. According to Eq. (1), if a surgical mask is being used to cover a N95 FFR, the length of the porous medium (ie, thickness of both masks) is increased and therefore a lower air flow rate penetrates through the masks for the same pressure drop across the masks. To maintain a normal minute ventilation, the breathing pressure (pressure drop, *Δp*) must therefore increase as the resistance increases.

Considering Eq. (1), if Q_B_ is the normal volumetric flow rate passing through a mask, then the pressure drop across this mask is defined as follows:(2)
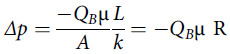



where *R* is defined as flow resistance (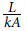), analogous to a resistor in conduction of electricity.^7^

For multiple masks (ie, a surgical mask over a N95 FFR), the total pressure drop across the masks is equal to the sum of the pressure drop across each mask individually.

Therefore, the total pressure drop can be defined as follows:(3)
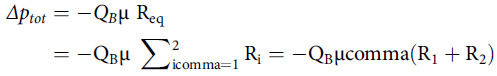



where *R*_*eq*_ is the equivalent flow resistance of the 2 masks and *R*_1_ and *R*_2_ are the flow resistances for the N95 FFR and the surgical mask, respectively. Equivalent flow resistance is calculated by the summation of resistances, similar to resistors in series in an electrical circuit.^7^ From Eq. (3), because the breathing flow rate is unchanged and the flow resistance has increased by the amount of *R*_2_, the total pressure drop across the 2 masks increases as compared to only using an N95 FFR. This additional resistance from the addition of the surgical mask, in turn, creates increased breathing pressures within the mask and airway relative to atmospheric (room air) pressure when the user maintains normal minute ventilation.

Thus, the respiratory cycle pressures will necessarily be more negative on inspiration and more positive on expiration to overcome the increased resistance of the combined masks in an attempt to maintain normal airflow or minute ventilation. As the pressure drop increases across the masks, the same atmospheric-to-airway pressure drop applies to the N95 FFR edge-to-face seal. As a result, as higher pressure differentials pulsate across a pliable mechanical seal, such as the N95 FFR edge-to-face seal, leakage can incrementally occur.

When the face and N95 FFR edge meet to form a seal, any separation between the mating surfaces increases leakage substantially. Doubling seal-surface separation can increase leakage by a factor of 8.^8^ This is shown by approximating the critical constriction at a seal interface as a pore with a rectangular cross section with a long width and relatively small height. Assuming incompressible Newtonian fluid, and *u*_1_ as the average height separating the mating surfaces, the volume flow per unit time, *Q_l_* (leakage flow rate), through the critical constriction is given by the following equation (Poiseuille flow):(4)
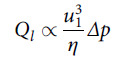



where η is the fluid viscosity, and Δ*p* is the pressure differential across the masks as shown in Eq. (3).^8^

## Results

The increased pulsating pressure differential created by an overlying surgical mask potentially causes increased leakage according to Eq. (4).^8,9^

## Discussion

The analytical model includes simplifying assumptions such as negligible effects of multiphase flow and leakage around the surgical mask edges. In addition, the seal’s balance ratio might increase during inhalation, thereby creating a countering increase in seal competence.

Covering N95 FFRs with a surgical mask can potentially increase the occurrence of N95 FFR leakage. Appropriate assessment of this risk will require additional research, including higher-order theoretical analysis, computational fluid dynamics modeling, bench tests, and/or human studies. As we engage in that work, we encourage others to do the same. Pending further study, N95 FFR clinical guidance and instructions to cover N95 FFRs with surgical masks should consider and assess this risk.

## References

[1] Tran K , Cimon K , Severn M , Pessoa-Silva CL , Conly J. Aerosol-generating procedures and risk of transmission of acute respiratory infections to healthcare workers: a systematic review. PLoS One 2012;7(4):e35797. 22563403 10.1371/journal.pone.0035797 PMC3338532

[2] Interim infection prevention and control recommendations for healthcare personnel during the coronavirus disease 2019 (COVID-19) pandemic. Centers for Disease Control and Prevention website. https://www.cdc.gov/coronavirus/2019-ncov/hcp/infection-control-recommendations.html. Published 2020. Updated July 15, 2020. Accessed August 12, 2020.

[3] Bartoszko JJ , Farooqi MAM , Alhazzani W , Loeb M. Medical masks vs N95 respirators for preventing COVID-19 in healthcare workers: a systematic review and meta-analysis of randomized trials. Influenza Other Respir Viruses 2020;14:365–373. 32246890 10.1111/irv.12745 PMC7298295

[4] Czubryt MP , Stecy T , Popke E , et al. N95 Mask reuse in a major urban hospital—COVID-19 response process and procedure. J Hosp Infect 2020:106:277–282. 32745590 10.1016/j.jhin.2020.07.035 PMC7837009

[5] Whitaker S. Flow in porous media—a theoretical derivation of Darcy’s Law. Transport Porous Med 1986;1(1):3–25.

[6] Nishiyama N , Yokoyama T. Permeability of porous media: role of the critical pore size. J Geophys Res-Sol Ea 2017;122:6955–6971.

[7] Hagen KD. Heat Transfer with Applications. Upper Saddle River, NJ: Prentice Hall; 1999.

[8] Lorenz B , Persson BNJ. Leak rate of seals: comparison of theory with experiment. Epl-Europhys Lett 2009;86(4):44006.

[9] Persson BNJ , Yang C. Theory of the leak-rate of seals. J Phys-Condens Mat 2008;20(31):315011.

